# Supplementary material for: The relationship between congenital heart disease and cancer in Swedish children: A population-based cohort study
Source: PLoS Med. 2022 Feb 25;19(2):e1003903. doi: 10.1371/journal.pmed.1003903 (PMC8880823; doi:10.1371/journal.pmed.1003903)
Supplement: S1 Analysis Plan — (DOCX) [file pmed.1003903.s002.docx]

# PRELIMINARY TITLE:

Cancer risk in children with congenital heart disease: 40-year experience from Sweden.

# Aim & Study questions:

This study aims to determine the relationship between congenital heart disease (CHD) and malignancies in Swedish children and adolescents. We aim to answer the following research questions:

1. Do Swedish children and adolescents (0-19 years old) with a CHD diagnosis have a higher cancer risk compared to their counterparts free of the disease?
2. How is cancer subtype risk distributed in Swedish individuals of these age groups with a CHD diagnosis compared to the general population?

Should a predisposition to malignancy in patients with a CHD diagnosis be confirmed, it would entail broad implications about the study of CHD, the treatment and care of CHD patients, medical decision-making, and the potentially escalating merits of cancer surveillance. In view of the increasing patient population with CHD after improvements in their life expectancies, such insights can be particularly compelling.

# Background:

Birth defects are defined as “structural changes present at birth that can affect almost any part or parts of the body” [1], affect about 5-7% of newborns [2], and are the leading cause in infant mortality [3]. They also have a profound impact on live-born children, causing an array of long-term physical, physiologic or developmental disabilities [3]. Several studies have explored the relationship between birth defects and childhood cancer risk, with current evidence pointing toward a positive association with pediatric malignancies; if the aforementioned associations are indeed causal, a leading theory concerning a potential biological pathway is that a common genetic abnormality impairing normal development is the predisposing force toward both birth defects and subsequent malignancy [4].

CHD, customarily defined as “a structural abnormality of the heart or intrathoracic vessels present at birth that is actually or potentially of functional significance” [5], constitutes the most common birth defect in humans; it occurs in approximately 1% of all births [6]. In recent years, the prevalence of CHD has increased due to the advances in pediatric care and surgical therapies [7]; thus, children with CHD are increasingly living into adulthood [6]. Contrary to birth defects as a whole, however, there is limited research regarding the relationship between CHD and cancer risk; prospective, large-scale studies with long follow-up periods are especially sparse [8]. Nevertheless, current evidence suggests a potentially elevated risk for malignancies in patients with a diagnosis of CHD [6, 8].

# Methods:

The proposed study is a registry-based nationwide cohort conducted in Sweden. All children registered in the Swedish Medical Birth Register (Medicinska födelseregistret) with information on both biological parents will be included. The Medical Birth Register was established in 1973 and comprises data on practically all deliveries in Sweden [9]. The Medical Birth Register will be linked to the National Cancer Register (Cancerregistret) by using the unique personal identity number to retrieve cancer diagnoses. The Swedish Cancer Register was established in 1958 and covers the whole population [10]. Children enrolled in the study will be followed from birth until one of the following events occurs: cancer diagnosis, death, emigration, 20th birthday, or end of follow up (end of December 2015).

Individuals will be considered as suffering from CHD if they have been assigned at least one diagnostic code for CHD in the Medical Birth Register (adhering to the most recent ICD classification available at the time of diagnosis). Diagnoses from the National Patient Register (Patientregistret) will also be considered in order to capture CHD cases not diagnosed at birth, provided they predate any cancer detection by at least 2 years (different lag periods will be considered as a sensitivity analysis: 1 year, 6 months, 1 day). Regarding the outcome of interest, incident cancer is defined as the first diagnosis of primary cancer in the National Cancer Register during the observation period (classified according to the International Classification of Childhood Cancer, 3d edition).

Regarding the planned analyses, descriptive statistics will be used to report the baseline characteristics of the study cohort. Cox proportional hazards models will be used to estimate the effect of CHD on the risk of malignancies (childhood cancer overall and most common subtypes: leukemia, lymphoma, and CNS tumors). After calculating the crude relative risks, analyses will be adjusted for potential confounding variables: sex, birth decade, maternal/paternal age, maternal/paternal education, region of residence at birth, and genetic syndromes predisposing to certain tumors (phakomatoses, Down syndrome). Continuous covariates will be categorized using standard cut-off points. The analyses will be repeated in diagnoses from the Medical Birth Register only, so as to highlight any major differences arising from restricting the CHD diagnosis thusly. Part of the data (from 1982 onwards) will be also adjusted for maternal smoking, in a sensitivity analysis.

# References:

1. Facts about Birth Defects [Internet]. Centers for Disease Control and Prevention. Available from: https://www.cdc.gov/ncbddd/birthdefects/facts.html

2. Rosenberg, Leon;Rosenberg, Diane Drobnis. Human Genes and Genomes. Elsevier Science & Technology; 2012.

3. Tulchinsky TH, Orenstein WA. Case studies in public health. London ; San Diego, CA: Elsevier/Academic Press; 2018.

4. Johnson K, Lee J, Ahsan K, Padda H, Feng Q, Partap S et al. Pediatric cancer risk in association with birth defects: A systematic review. PLOS ONE. 2017;12(7).

5. McCusker C, Casey F. Congenital heart disease and neurodevelopment : understanding and improving outcomes. Amsterdam: Elsevier/Academic Press; 2016.

6. Collins R, Von Behren J, Yang W, Carmichael S, Reynolds P, Fisher P et al. Congenital heart disease complexity and childhood cancer risk. Birth Defects Research. 2018;110(17):1314-1321.

7. Mandalenakis Z, Rosengren A, Skoglund K, Lappas G, Eriksson P, Dellborg M. Survivorship in Children and Young Adults With Congenital Heart Disease in Sweden. JAMA Internal Medicine. 2017;177(2):224.

8. Lee Y, Chen Y, Jeng M, Tsao P, Yen H, Lee P et al. The Risk of Cancer in Patients with Congenital Heart Disease: A Nationwide Population-Based Cohort Study in Taiwan. PLOS ONE. 2015;10(2):e0116844.

9. Källén B, Källén K. The Swedish Medical Birth Register - a summary of content and quality. Socialstyrelsen, 2003.

10. Barlow L, Westergren K, Holmberg L, Talback M. The completeness of the Swedish Cancer Register: a sample survey for year 1998. Acta Oncol. 2009;48(1):27-33. Epub 2008/09/04. doi: 10.1080/02841860802247664. PubMed PMID: 18767000.
