# Supplementary figures and images for: The relationship between congenital heart disease and cancer in Swedish children: A population-based cohort study
Source: PLoS Med. 2022 Feb 25;19(2):e1003903. doi: 10.1371/journal.pmed.1003903 (PMC8880823; doi:10.1371/journal.pmed.1003903)

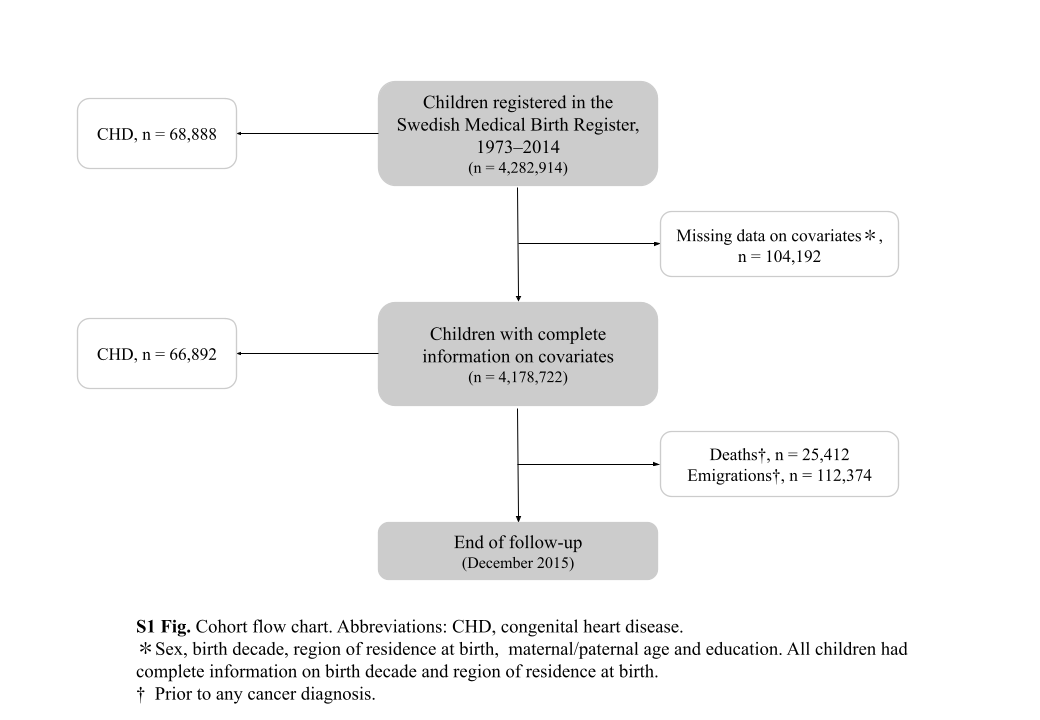

Supplement: S1 Fig — (PNG) [file pmed.1003903.s003.png]
