# Supplementary material for: The relationship between congenital heart disease and cancer in Swedish children: A population-based cohort study
Source: PLoS Med. 2022 Feb 25;19(2):e1003903. doi: 10.1371/journal.pmed.1003903 (PMC8880823; doi:10.1371/journal.pmed.1003903)
Supplement: S1 Table — (DOCX) [file pmed.1003903.s004.docx]

| **S1 Table. List of ICD-10 codes and their representative lesions evaluated as severe.** | |
| --- | --- |
| **ICD-10** | **Code description** |
| **Q20** |  |
| Q20.0 | Common truncus, Persistent truncus arteriosus |
| Q20.1 | Double outlet right ventricle |
| Q20.2 | Double outlet left ventricle |
| Q20.3 | Discordant ventriculoarterial connection, Transposition of the great vessels |
| Q20.4 | Double inlet ventricle |
| **Q22** |  |
| Q22.0 | Pulmonary valve atresia |
| Q22.4 | Congenital tricuspid stenosis, Tricuspid atresia |
| Q22.6 | Hypoplastic right heart syndrome |
| **Q23** |  |
| Q23.2 | Congenital mitral stenosis, Congenital mitral atresia |
| Q23.4 | Hypoplastic left heart syndrome |
| **Q25** |  |
| Q25.2 | Atresia of aorta |
| Q25.5 | Atresia of pulmonary artery |
| Any cyanotic congenital heart disease was also evaluated as severe. All other codes in Q20.0–Q26.9, Q89.3 were considered as mild-moderate.  The ICD-10 codes were back-translated to ICD-9 and ICD-8. | |
