# Supplementary material for: The relationship between congenital heart disease and cancer in Swedish children: A population-based cohort study
Source: PLoS Med. 2022 Feb 25;19(2):e1003903. doi: 10.1371/journal.pmed.1003903 (PMC8880823; doi:10.1371/journal.pmed.1003903)
Supplement: S2 Table — CHD, congenital heart disease; CNS, central nervous system. (DOCX) [file pmed.1003903.s005.docx]

| **S2 Table. Distribution of exposure and main outcomes in the study population, according to completeness of data on the main covariates.** | | |
| --- | --- | --- |
|  | **Individuals with complete**  **information on covariates**  **(N= 4,178,722; 97.6%)** | **Individuals with missing values on covariates**  **N=104,192; 2.4%)** |
|  | **n (%)** | **n (%)** |
| **CHD** |  |  |
| No CHD | 4,111,830 (98.4) | 102,196 (98.0) |
| Mild-Moderate | 62,163 (1.5) | 1,714 (1.7) |
| Severe | 4,729 (0.1) | 282 (0.3) |
| **Childhood cancer** | 10,911 (0.3) | 180 (0.2) |
| CNS | 2,900 (0.1) | 50 (0.1) |
| Leukemia | 2,967 (0.1) | 57 (0.1) |
| Lymphoma | 1,273 (<0.1) | 8 (<0.1) |
| Hepatoblastoma | 122 (<0.1) | 1 (<0.1) |
| Neuroblastoma | 401 (<0.1) | 18 (<0.1) |
| **Abbreviations:**  CHD , Congenital Heart Disease ; CNS , central nervous system. | | |
