# Supplementary material for: The relationship between congenital heart disease and cancer in Swedish children: A population-based cohort study
Source: PLoS Med. 2022 Feb 25;19(2):e1003903. doi: 10.1371/journal.pmed.1003903 (PMC8880823; doi:10.1371/journal.pmed.1003903)
Supplement: S3 Table — CHD, congenital heart disease; CI, confidence interval; CNS, central nervous system; HR, hazard ratio. (DOCX) [file pmed.1003903.s006.docx]

| **S3 Table. Adjusted HRs (95% CIs) of cancer in children with Congenital Heart Disease (CHD) born from 1982 onwards.** | | |
| --- | --- | --- |
|  | **CHD/ No CHD**  **no. of cases** | **HR (95% CI)** |
| **Total cancers** | 174/7,283 | 1.01 (0.86–1.19) |
| **CNS** | 24/1,921 | 0.66 (0.44–0.99) |
| **Leukemia** | 73/2,040 | 0.83 (0.63–1.10) |
| **Lymphoma** | 18/773 | 1.63 (1.02–2.60) |
| **Hepatoblastoma** | 4/92 | 2.63 (0.97–7.17) |
| **Neuroblastoma** | 7/344 | 1.13 (0.52–2.43) |
| **A**djusted for birth decade, maternal/paternal age and education, region of residence at birth, neurocutaneous syndromes, Down syndrome, maternal smoking  **Abbreviations:**  HR , hazard ratio ; CI , confidence interval ; CHD , congenital heart disease ; CNS , central nervous system. | | |
