# Supplementary material for: The relationship between congenital heart disease and cancer in Swedish children: A population-based cohort study
Source: PLoS Med. 2022 Feb 25;19(2):e1003903. doi: 10.1371/journal.pmed.1003903 (PMC8880823; doi:10.1371/journal.pmed.1003903)
Supplement: S4 Table — CHD, congenital heart disease; CI, confidence interval; CNS, central nervous system; HR, hazard ratio. (DOCX) [file pmed.1003903.s007.docx]

| **S4 Table. Adjusted HRs (95% CIs) of cancer in children with Congenital Heart Disease (CHD) born from 1987 onwards.** | | | | |
| --- | --- | --- | --- | --- |
|  | **CHD/ No CHD**  **no. of cases** | **MODEL 1**  **HR (95% CI)** | **MODEL 2**  **HR (95% CI)** | **MODEL 3**  **HR (95% CI)** |
| **Total cancers** | 164/6,355 | 1.53 (1.31–1.78) | 1.46 (1.25–1.71) | 1.01 (0.85–1.20) |
| **CNS** | 24/1,619 | 0.88 (0.59–1.32) | 0.76 (0.51–1.15) | 0.73 (0.48–1.10) |
| **Leukemia** | 70/1,819 | 2.20 (1.73–2.79) | 2.20 (1.73–2.79) | 0.82 (0.61–1.09) |
| **Lymphoma** | 17/621 | 1.71 (1.06–2.78) | 1.70 (1.06–2.76) | 1.77 (1.10–2.87) |
| **Model 1:** adjusted for birth decade, maternal/paternal age and education, region of residence at birth  **Model 2:** adjusted for birth decade, maternal/paternal age and education, region of residence at birth, neurocutaneous syndromes  **Model 3:** adjusted for birth decade, maternal/paternal age and education, region of residence at birth, neurocutaneous syndromes, Down syndrome  **Abbreviations:**  HR , hazard ratio ; CI , confidence interval ; CHD , congenital heart disease ; CNS , central nervous system. | | | | |
