# Supplementary material for: The relationship between congenital heart disease and cancer in Swedish children: A population-based cohort study
Source: PLoS Med. 2022 Feb 25;19(2):e1003903. doi: 10.1371/journal.pmed.1003903 (PMC8880823; doi:10.1371/journal.pmed.1003903)
Supplement: S5 Table — CHD, congenital heart disease; CI, confidence interval; CNS, central nervous system; HR, hazard ratio. (DOCX) [file pmed.1003903.s008.docx]

| **S5 Table. Adjusted HRs (95% CIs) of cancer in children with congenital heart disease (CHD)**— **different lag periods between CHD diagnosis from the Patient Register and cancer diagnosis.** | | | | |
| --- | --- | --- | --- | --- |
|  | **CHD/ No CHD**  **no. of cases** | **MODEL 1**  **HR (95% CI)** | **MODEL 2**  **HR (95% CI)** | **MODEL 3**  **HR (95% CI)** |
| **CHD diagnosis at least 1 year before a cancer diagnosis** | | | | |
| **Total cancers** | 235/10,676 | 1.59 (1.40–1.81) | 1.52 (1.33–1.73) | 1.13 (0.99–1.31) |
| Males | 110/5,749 | 1.43 (1.18–1.73) | 1.36 (1.13–1.65) | 1.04 (0.85–1.27) |
| Females | 125/4,927 | 1.77 (1.48–2.11) | 1.68 (1.41–2.01) | 1.24 (1.02–1.51) |
| **CNS** | 37/2,863 | 0.95 (0.69–1.31) | 0.82 (0.60–1.14) | 0.82 (0.59–1.14) |
| Males | 17/1,490 | 0.86 (0.53–1.39) | 0.74 (0.46–1.20) | 0.72 (0.45–1.18) |
| Females | 20/1,373 | 1.04 (0.67–1.62) | 0.91 (0.58–1.42) | 0.92 (0.59–1.44) |
| **Leukemia** | 99/2,868 | 2.38 (1.94–2.90) | 2.37 (1.94–2.90) | 1.01 (0.80–1.28) |
| Males | 40/1,584 | 1.80 (1.31–2.46) | 1.80 (1.32–2.47) | 0.80 (0.56–1.15) |
| Females | 59/1,284 | 3.03 (2.33–3.94) | 3.01 (2.32–3.92) | 1.22 (0.89–1.69) |
| **Lymphoma** | 26/1,247 | 1.66 (1.13–2.45) | 1.65 (1.12–2.44) | 1.72 (1.17–2.54) |
| Males | 19/776 | 1.97 (1.25–3.11) | 1.97 (1.25–3.11) | 2.05 (1.30–3.24) |
| Females | 7/471 | 1.16 (0.55–2.45) | 1.15 (0.54–2.42) | 1.19 (0.57–2.52) |
| **Hepatoblastoma** | 7/115 | 3.77 (1.75–8.11) | 3.78 (1.76–8.12) | 3.95 (1.84–8.49) |
| Males | 4/68 | 3.71 (1.35–10.20) | 3.72 (1.35–10.22) | 3.88 (1.41–10.67) |
| Females | 3/47 | 3.89 (1.21–12.56) | 3.90 (1.21–12.58) | 4.08 (1.26–13.16) |
| **Neuroblastoma** | 9/392 | 1.39 (0.72–2.68) | 1.38 (0.71–2.67) | 1.34 (0.68–2.63) |
| Males | 5/219 | 1.44 (0.59–3.50) | 1.43 (0.59–3.47) | 1.32 (0.52–3.30) |
| Females | 4/173 | 1.32 (0.49–3.57) | 1.33 (0.49–3.57) | 1.38 (0.51–3.72) |
| **CHD diagnosis at least 6 months before a cancer diagnosis** | | | | |
| **Total cancers** | 237/10,674 | 1.60 (1.41–1.82) | 1.53 (1.35–1.74) | 1.15 (1.00–1.32) |
| Males | 112/5,747 | 1.46 (1.21–1.76) | 1.39 (1.15–1.68) | 1.06 (0.87–1.30) |
| Females | 125/4,927 | 1.77 (1.48–2.11) | 1.68 (1.41–2.01) | 1.24 (1.02–1.51) |
| **CNS** | 38/2,862 | 0.98 (0.71–1.35) | 0.85 (0.61–1.17) | 0.84 (0.61–1.17) |
| Males | 18/1,489 | 0.91 (0.57–1.45) | 0.79 (0.49–1.26) | 0.77 (0.48–1.23) |
| Females | 20/1,373 | 1.04 (0.67–1.62) | 0.91 (0.58–1.42) | 0.92 (0.59–1.44) |
| **Leukemia** | 99/2,868 | 2.38 (1.94–2.90) | 2.37 (1.94–2.90) | 1.01 (0.80–1.28) |
| Males | 40/1,584 | 1.80 (1.31–2.46) | 1.80 (1.32–2.47) | 0.80 (0.56–1.15) |
| Females | 59/1,284 | 3.03 (2.33–3.94) | 3.01 (2.32–3.92) | 1.22 (0.89–1.69) |
| **Lymphoma** | 26/1,247 | 1.66 (1.13–2.45) | 1.65 (1.12–2.44) | 1.72 (1.17–2.54) |
| Males | 19/776 | 1.97 (1.25–3.11) | 1.97 (1.25–3.11) | 2.05 (1.30–3.24) |
| Females | 7/471 | 1.16 (0.55–2.45) | 1.15 (0.54–2.42) | 1.19 (0.57–2.52) |
| **Hepatoblastoma** | 8/114 | 4.35 (2.12–8.94) | 4.36 (2.12–8.95) | 4.56 (2.22–9.35) |
| Males | 5/67 | 4.72 (1.89–11.73) | 4.72 (1.90–11.75) | 4.93 (1.98–12.27) |
| Females | 3/47 | 3.89 (1.21–12.56) | 3.90 (1.21–12.58) | 4.08 (1.26–13.16) |
| **Neuroblastoma** | 9/392 | 1.39 (0.72–2.68) | 1.38 (0.71–2.67) | 1.34 (0.68–2.63) |
| Males | 5/219 | 1.44 (0.59–3.50) | 1.43 (0.59–3.47) | 1.32 (0.52–3.30) |
| Females | 4/173 | 1.32 (0.49–3.57) | 1.33 (0.49–3.57) | 1.38 (0.51–3.72) |
| **CHD diagnosis at least 1 day before a cancer diagnosis** | | | | |
| **Total cancers** | 262/10,649 | 1.78 (1.57–2.01) | 1.70 (1.50–1.92) | 1.30 (1.13–1.48) |
| Males | 126/5,733 | 1.64 (1.38–1.96) | 1.57 (1.31–1.87) | 1.22 (1.01–1.47) |
| Females | 136/4,916 | 1.93 (1.62–2.28) | 1.84 (1.55–2.18) | 1.38 (1.14–1.66) |
| **CNS** | 41/2,859 | 1.05 (0.77–1.44) | 0.92 (0.67–1.25) | 0.91 (0.67–1.25) |
| Males | 19/1,488 | 0.96 (0.61–1.52) | 0.83 (0.53–1.31) | 0.81 (0.51–1.29) |
| Females | 22/1,371 | 1.15 (0.75–1.75) | 1.00 (0.66–1.53) | 1.02 (0.67–1.56) |
| **Leukemia** | 111/2,856 | 2.68 (2.21–3.24) | 2.67 (2.21–3.23) | 1.20 (0.96–1.50) |
| Males | 47/1,577 | 2.13 (1.59–2.84) | 2.12 (1.59–2.85) | 1.00 (0.71–1.39) |
| Females | 64/1,279 | 3.30 (2.57–4.25) | 3.28 (2.55–4.23) | 1.40 (1.02–1.91) |
| **Lymphoma** | 28/1,245 | 1.79 (1.23–2.61) | 1.78 (1.22–2.59) | 1.86 (1.27–2.70) |
| Males | 21/774 | 2.19 (1.42–3.38) | 2.18 (1.42–3.37) | 2.28 (1.48–3.52) |
| Females | 7/471 | 1.16 (0.55–2.45) | 1.15 (0.54–2.42) | 1.19 (0.57–2.52) |
| **Hepatoblastoma** | 8/114 | 4.35 (2.12–8.94) | 4.36 (2.12–8.95) | 4.56 (2.22–9.35) |
| Males | 5/67 | 4.72 (1.89–11.73) | 4.72 (1.90–11.75) | 4.93 (1.98–12.27) |
| Females | 3/47 | 3.89 (1.21–12.56) | 3.90 (1.21–12.58) | 4.08 (1.26–13.16) |
| **Neuroblastoma** | 14/387 | 2.19 (1.28–3.73) | 2.18 (1.28–3.71) | 2.15 (1.25–3.71) |
| Males | 7/217 | 2.04 (0.96–4.33) | 2.02 (0.95–4.29) | 1.90 (0.87–4.15) |
| Females | 7/170 | 2.36 (1.11–5.03) | 2.36 (1.11–5.04) | 2.46 (1.15–5.24) |
| **CHD diagnosis at least 2 years before/after a cancer diagnosis** | | | | |
| **Total cancers** | 273/10,638 | 1.85 (1.64–2.09) | 1.77 (1.57–2.00) | 1.36 (1.20–1.55) |
| Males | 125/5,734 | 1.63 (1.36–1.94) | 1.56 (1.30–1.86) | 1.21 (1.00–1.46) |
| Females | 148/4,904 | 2.10 (1.78–2.48) | 2.00 (1.70–2.36) | 1.53 (1.28–1.82) |
| **CNS** | 39/2,861 | 1.00 (0.73–1.38) | 0.87 (0.63–1.19) | 0.87 (0.63–1.19) |
| Males | 17/1,490 | 0.86 (0.53–1.39) | 0.74 (0.46–1.20) | 0.72 (0.45–1.17) |
| Females | 22/1,371 | 1.15 (0.75–1.75) | 1.00 (0.66–1.53) | 1.02 (0.67–1.56) |
| **Leukemia** | 117/2,850 | 2.83 (2.35–3.40) | 2.82 (2.34–3.39) | 1.29 (1.03–1.61) |
| Males | 50/1,574 | 2.27 (1.71–3.00) | 2.27 (1.71–3.01) | 1.09 (0.78–1.50) |
| Females | 67/1,276 | 3.47 (2.71–4.44) | 3.44 (2.69–4.41) | 1.50 (1.11–2.04) |
| **Lymphoma** | 29/1,244 | 1.86 (1.28–2.68) | 1.85 (1.28–2.67) | 1.92 (1.33–2.78) |
| Males | 20/775 | 2.08 (1.33–3.24) | 2.08 (1.33–3.24) | 2.17 (1.39–3.38) |
| Females | 9/469 | 1.50 (0.77–2.90) | 1.48 (0.77–2.87) | 1.54 (0.80–2.98) |
| **Hepatoblastoma** | 11/111 | 6.16 (3.31–11.48) | 6.16 (3.31–11.49) | 6.45 (3.46–12.02) |
| Males | 4/68 | 3.71 (1.35–10.20) | 3.72 (1.35–10.21) | 3.88 (1.41–10.66) |
| Females | 7/43 | 10.02 (4.48–22.41) | 10.03 (4.49–22.44) | 10.50 (4.69–23.47) |
| **Neuroblastoma** | 11/390 | 1.70 (0.93–3.10) | 1.70 (0.93–3.09) | 1.66 (0.90–3.06) |
| Males | 6/218 | 1.74 (0.77–3.91) | 1.72 (0.76–3.88) | 1.61 (0.69–3.72) |
| Females | 5/172 | 1.66 (0.68–4.05) | 1.67 (0.68–4.06) | 1.73 (0.71–4.22) |
| **Model 1:** adjusted for birth decade, maternal/paternal age and education, region of residence at birth  **Model 2:** adjusted for birth decade, maternal/paternal age and education, region of residence at birth, neurocutaneous syndromes  **Model 3:** adjusted for birth decade, maternal/paternal age and education, region of residence at birth, neurocutaneous syndromes, Down syndrome  **Abbreviations:**  HR , hazard ratio ; CI , confidence interval ; CHD , congenital heart disease ; CNS , central nervous system. | | | | |
