# Supplementary material for: The relationship between congenital heart disease and cancer in Swedish children: A population-based cohort study
Source: PLoS Med. 2022 Feb 25;19(2):e1003903. doi: 10.1371/journal.pmed.1003903 (PMC8880823; doi:10.1371/journal.pmed.1003903)
Supplement: S6 Table — CHD, congenital heart disease; CI, confidence interval; CNS, central nervous system; HR, hazard ratio; MBR, Medical Birth Register. (DOCX) [file pmed.1003903.s009.docx]

| **S6 Table. Adjusted HRs (95% CIs) of cancer in children with Congenital Heart Disease (CHD) diagnosed from the Medical Birth Register (MBR).** | | | | |
| --- | --- | --- | --- | --- |
|  | **CHD/ No CHD**  **no. of cases** | **MODEL 1**  **HR (95% CI)** | **MODEL 2**  **HR (95% CI)** | **MODEL 3**  **HR (95% CI)** |
| **Total cancers** | 155/10,756 | 1.95 (1.66–2.28) | 1.88 (1.60–2.20) | 1.45 (1.23–1.71) |
| Males | 71/5,788 | 1.74 (1.38–2.20) | 1.68 (1.33–2.12) | 1.31 (1.03–1.67) |
| Females | 84/4,968 | 2.16 (1.74–2.68) | 2.09 (1.68–2.59) | 1.59 (1.27–1.99) |
| **CNS** | 25/2,875 | 1.18 (0.80–1.75) | 1.06 (0.71–1.57) | 1.05 (0.71–1.57) |
| Males | 12/1,495 | 1.15 (0.65–2.03) | 1.01 (0.57–1.79) | 1.00 (0.56–1.77) |
| Females | 13/1,380 | 1.21 (0.70–2.09) | 1.09 (0.63–1.89) | 1.11 (0.64–1.92) |
| **Leukemia** | 68/2,899 | 3.12 (2.45–3.96) | 3.11 (2.45–3.96) | 1.41 (1.08–1.84) |
| Males | 29/1,595 | 2.52 (1.75–3.64) | 2.53 (1.75–3.65) | 1.20 (0.80–1.79) |
| Females | 39/1,304 | 3.79 (2.75–5.21) | 3.77 (2.74–5.18) | 1.62 (1.13–2.32) |
| **Lymphoma** | 10/1,263 | 1.11 (0.60–2.07) | 1.10 (0.59–2.06) | 1.15 (0.62–2.14) |
| Males | 7/788 | 1.29 (0.61–2.72) | 1.29 (0.61–2.71) | 1.34 (0.64–2.82) |
| Females | 3/475 | 0.83 (0.27–2.60) | 0.83 (0.27–2.57) | 0.86 (0.27–2.66) |
| **Model 1:** adjusted for birth decade, maternal/paternal age and education, region of residence at birth  **Model 2:** adjusted for birth decade, maternal/paternal age and education, region of residence at birth, neurocutaneous syndromes  **Model 3:** adjusted for birth decade, maternal/paternal age and education, region of residence at birth, neurocutaneous syndromes, Down syndrome  **Abbreviations:**  HR , hazard ratio ; CI , confidence interval ; CHD , congenital heart disease ; MBR , Medical Birth Register ; CNS , central nervous system. | | | | |
