# Supplementary material for: The relationship between congenital heart disease and cancer in Swedish children: A population-based cohort study
Source: PLoS Med. 2022 Feb 25;19(2):e1003903. doi: 10.1371/journal.pmed.1003903 (PMC8880823; doi:10.1371/journal.pmed.1003903)
Supplement: Attachment — Submitted filename: PLOS_Review_CHD and Childhood Cancer risk among Swedish Children_20210805.docx [file pmed.1003903.s010.docx]

**Summary:** This is a retrospective population-based cohort study conducted in order to determine the association between CHD and childhood cancer incidence among Swedish children. Using Medical Birth Registry and National Patient Register, the study identified over 68,000 CHD patients among nearly 4,171,000 newborn children between 1973 to 2014. Childhood cancers were retrieved by linking Swedish Cancer Registries. The associations were assessed using Cox’s proportional hazards model adjusting for birth decade, birth weight for gestational age, maternal/paternal age and education, region of residence at birth, neurocutaneous syndromes and/or Down syndrome.

**Overall comments:** Several other studies reported an elevated risk of cancer among both children and adults with CHD. However, the underlying mechanism is poorly understood. This study also provided evidence in favour of increased childhood cancer risk among children with CHD but as such, did not provide significant new information.

The strength of the study is the nationally representative large database spanning over 40 years of observation period. The objective of the study is clearly mentioned. However, with multiple sub-analyses, sensitivity analyses and outcomes, the communication and interpretability of results necessarily becomes challenging for the readers to follow.

Mentioning general and specific objectives with support of rationale could have been helpful. The study design and statistical methods are appropriate. However, there are some major concerns that need to be addressed:

1. **Potential immortal time bias?** The individuals not diagnosed with CHD at birth were identified as CHD (exposed) using National Patient Register (NPR) on a later date after birth. Since the authors followed the final study population from birth, the time between the cohort entry and CHD diagnosis became ‘immortal’ for the exposed children; they cannot have cancer by design to have being classified as exposed. Did the author consider time-dependent exposure definition in this regard?
2. **2-year lag time:** The authors used 2 years of lag time for the subjects who were not diagnosed as a CHD patient at birth in order to account for surveillance bias. How about for the patients who were diagnosed at birth? Did they also apply lag time for them? If not, what were the assumptions and how are they not subject to such bias?
3. **Adjusting for competing risk of death:** The analyses did not account for competing risk of death. Even though the mortality rate might be lower in this pediatric population, adjusting for death might account for overestimation of the association reported.
4. **Adjusting for CHD severity:** The authors presented CHD severity-stratified analyses but did not account for it in other models. More specifically, the individuals diagnosed as CHD patients at births are those with more severe lesions. Not controlling for them in the combined cohort might represent a spurious association.
5. **Dealing with Missing data:** The authors excluded individuals with missing covariates (2.6%). However, they did not give any detail on the characteristics of these missing subjects: Are they more severe than those included in the final cohort or otherwise? What is the distribution of the primary endpoints among them? Are they risking any selection bias?
6. **Medical Comorbidities:** The authors did not report or adjusted for any medical comorbidities. More severe CHD patients might have more medical comorbidities and would lead to increased surveillance. Do the authors consider adjusting for them?
7. **Sex as EMM or Confounder:** The authors adjusted for Sex in their models and also reported the sex-stratified hazard ratios. This reviewer does not find it informative in a situation when the manuscript did not discuss on the association by sex.
8. **Birth weight, Age categorized:** The authors represented and adjusted for birth weight for gestational age and maternal/paternal age as categorical variable. How did they choose the non-linear representation of the confounders over linear one? Did they perform any model comparison (eg. LRT)? They did not mention possibilities of residual confounding in this regard or any other sources (eg. omitting a very important confounder due to data limitation) in their discussion.
9. **Reporting:** This reviewer would like to mention some reporting issues that might be helpful to the authors:
   a. The authors might consider including a flowchart of derivation of the study population.
   b. Since this study relies on the time to event analyses, reporting follow-up times (median), total person-time etc. would be informative and helpful to understand the cohort.
   c. In Method section (Line 128), the author listed the potential confounders or *mediators*. From a theoretical and analytical point of view, we should not adjust for mediators. The authors might consider making correction for that.
   d. For binary variable (eg. yes/no), reporting proportions for both categories might seem redundant to some of the readers. The authors might consider reporting only one (eg. yes)
   e. In the discussion section, the authors mentioned that their sub-analyses by age at cancer diagnosis did not support the hypothesis that exposure to LDIR from cardiac procedure explains the CHD-Cancer association. However, the data were not provided. This reviewer finds it necessary to provide the data in support to their claim.
