# Supplementary material for: The relationship between congenital heart disease and cancer in Swedish children: A population-based cohort study
Source: PLoS Med. 2022 Feb 25;19(2):e1003903. doi: 10.1371/journal.pmed.1003903 (PMC8880823; doi:10.1371/journal.pmed.1003903)
Supplement: Attachment — Submitted filename: R2_CHDcancer_editorialreviewer comments with responses_f.docx [file pmed.1003903.s012.docx]

**COMMENTS FROM THE ACADEMIC EDITOR:**

The immortal bias is still a concern. The authors may want to perform additional sensitivity analyses to assess how much of their results could potentially be explained by this bias.

**Response:** We wish to thank the academic editor for this suggestion. We have now added an extra sensitivity analysis (in S5 table), where we consider CHD diagnoses from the National Patient Register (NPR) at least 2 years before or after a cancer diagnosis. This addition is described in detail in our response to reviewer 2 below.

**COMMENTS FROM THE REVIEWERS:**

**Reviewer #2:**

I received the authors' responses and found that they acknowledged the critical issues raised for the most part. However, some important issues are left unsolved. I, therefore, request a further revision on the following points:

**Response:** We thank the reviewer once more for the valuable comments in the previous review of the manuscript. We were very pleased to hear that they were overall satisfied with the revisions made after considering said comments.

1. I find the problem of exposure definition in this paper to be two-fold. Firstly, unlike acquired conditions, CHD is a condition that is present from birth, regardless of when this condition is 'diagnosed'. This study defines the exposure based on CHD diagnosis from patient registries in addition to birth registries. That could lead to bias if the diagnose date is considered the start date of CHD condition. In this study, a patient not 'diagnosed' with CHD at birth but later had a CHD diagnosis (in NPR) were classified 'exposed' since the diagnosis and "unexposed" between birth to the diagnosis. This is wrong. The patient should be classified as "exposed" since birth even though his/her CHD condition was diagnosed later.

**Response:** We thank the reviewer for this thoughtful and valid remark: we agree that splitting person-time after the CHD diagnosis would indeed be concerning. We wish to clarify, however, that this is not what we have done. All individuals who fulfilled the criteria to be classified as exposed, were considered exposed from birth. To clarify this in the manuscript, we have now added the following sentences in the Methods section:

*“As CHD is a condition present from birth regardless of when it is diagnosed, individuals with a diagnosis from the Patient Register who fulfilled the above criteria were considered exposed from birth.”* (Lines 125-127)

*“…with attained age as the underlying time scale.”* (Line 153)

Secondly, in response to my previous comment 1, the authors stated that they classified a patient as 'unexposed' if their cancer diagnosis precedes the CHD diagnosis (in NPR). This approach raised the second concern of misclassification on exposure. Indeed, according to this classification, the unexposed patient would survive less than the exposed patients, giving an artificial advantage to them (exposed patients). In this regard, I would like to refer this paper [Lévesque et al. 2010] to understand the problem under discussion better.

**Response:** We thank the reviewer for this important comment. The reason for including a lag period between the CHD and cancer diagnosis is that we expect that CHD will be almost universally identified in the cancer population, due to the increased medical monitoring that occurs because of the cancer diagnosis/symptoms. In the Nordic context, an increasing use of healthcare services has been found already 6-12 months before a cancer diagnosis (1, 2)—and naturally would continue well after the cancer diagnosis. As such, we believe that not accounting for the increased medical monitoring would lead to a differential misclassification of the exposure (i.e. children with cancer being more likely to be diagnosed with CHD than children without cancer), which would overestimate the risk of cancer in children with CHD.

To avoid this, we have introduced the lag periods. While our approach might lead to some underestimation of cancer risk in children with CHD (the children with cancer that had the first CHD diagnosis in proximity or after the cancer diagnosis will be classified as unexposed), we believe that this is better than the alternative. If we do not use these lag periods, a substantial differential misclassification of the exposure is likely to occur, because nearly all children with cancer and CHD will have a CHD diagnosis reported to the NPR, while a much smaller proportion of children with CHD (but without cancer) will have their CHD diagnosis reported to the NPR (as mild CHD might remain undetected or even be managed entirely by primary healthcare).

Thanks to the reviewer´s comment, we had the possibility to reflect more on our analyses and in order to minimize any underestimation introduced with this design, we have now decided to introduce a further criterion for CHD diagnoses from the NPR, only allowing them to occur before an individuals’ 20^th^ birthday (when we stop follow-up): because of this there are now fewer individuals that are classified as having CHD (66,892 instead of previously 68,943). In the previous analysis, children with cancer were classified as exposed only if the CHD diagnosis occurred before the cancer diagnosis, while for the individuals without cancer they could have been classified as exposed even if they had the first CHD diagnosis in their 30s. We realized that this may have introduced a differential exposure misclassification (individuals without cancer had a longer period of time in which we considered CHD diagnosis), which may have led to an underestimation of the association between CHD and cancer risk. To explain this update, we have added the following sentence in the methods section:

*“As the criterion introduced for considering diagnoses from the Patient Register might lead to individuals who do not develop cancer having more time to be diagnosed with CHD, we further restricted the CHD diagnosis up to age 19 (a deviation from our original analysis plan).”* (Lines 122-125)

Further, the reviewer raised the concern that children who had a CHD diagnosis after the cancer diagnosis were classified as unexposed. Therefore, we have introduced an sensitivity analysis that is reported in table S5. In this additional sensitivity analysis, we considered CHD diagnoses from the NPR at least 2 years before the cancer diagnosis (as in the main analysis), but in addition we also considered (first) CHD diagnoses that occurred more than two years after a cancer diagnosis. The rationale for this approach is that we expect that most of the increased medical monitoring due to the cancer diagnosis would occur only in the 2 years preceding and following the cancer diagnosis. As expected, estimates were higher in this sensitivity analysis and the increased leukemia risk was not entirely explained away by Down syndrome (although it was reduced quite substantially). As this analysis might still be a possible overestimation of the true childhood cancer risk (it is likely that for some individuals the increased medical monitoring would last longer than two years after cancer diagnosis), we would prefer to keep the conservative estimates of our main analysis as the main focus.

Regarding the issue of immortal time bias, the reviewer is suggesting that this could occur because individuals classified as exposed have an artificial advantage over the unexposed children, since they survived until their CHD diagnosis. However, we do not believe that this is a major issue in our study. Indeed, mortality in the pediatric population under study is particularly low, therefore children without cancer or other congenital malformations (including CHD) have a very low risk of death: therefore, mortality in the unexposed population is particularly small.

We expect that immortal time bias would have been a major issue if we wanted to study childhood cancer survival and used CHD diagnosis that occurred after a cancer diagnosis. In this example, we would have clearly introduced an immortal time period between the cancer diagnosis and the CHD diagnosis (that occurred after the cancer diagnosis). However, in our study we wanted to study childhood cancer risk and use diagnoses of CHD that occurred later in life (but before the cancer diagnosis) only to have a better exposure classification, since not all individuals with CHD are diagnosed at birth. We also think that immortal time bias is not an issue in the recently added sensitivity analysis (reported in table S5), in which we also considered CHD diagnosed two years after the cancer diagnosis. In this analysis, the person-time is still stopped at the time of the cancer diagnosis (and started at birth), and we are only using information after the cancer diagnosis to reclassify these individuals in a different exposure category.

2. As requested, the authors provided evidence supporting their claim against the hypothesis that exposure to LDIR from cardiac procedures explains the CHD-Cancer association. However, the evidence (S5 Table. Adjusted HRs (95% CIs) of cancer in children with Congenital Heart Disease (CHD), stratified by age at cancer diagnosis) is not convincing. The authors claim that solid tumors such as lymphomas typically need 10 to 15 years after radiation exposure to manifest. However, there still seems to be some uncertainty regarding the latency between low-dose ionization radiation exposures and cancer incidence. For example, in a large population study in Australia (Mathews, J. D., et al. Cancer risk in 680 000 people exposed to computed tomography scans in childhood or adolescence: data linkage study of 11 million Australians. Bmj (2013) 346.) that assessed the association between CT scan and cancer in children and adolescents, it was reported that " At 1-4, 5-9, 10-14, and 15 or more years since first exposure, IRRs were 1.35 (1.25 to 1.45), 1.25 (1.17 to 1.34), 1.14 (1.06 to 1.22), and 1.24 (1.14 to 1.34), respectively." Thus, simply comparing the CHD-cancer association between groups with age at cancer diagnosis before and after 10 years of age could not be used as evidence for assessing the LDIR-cancer association among CHD children population.

There is a knowledge base that says otherwise and is well established based on large epidemiological studies (refs). As in this study cohort, however, stated by the authors in lines 280-281, "8 of the 25 CHD patients with lymphoma had their CHD diagnosis <10 years before they were diagnosed with lymphoma" (with longer latency). This goes back to my first point; how did they define their exposure.

To summarize, the subgroup analysis by age at cancer diagnosis thus does not provide compelling evidence to claim that exposure to LDIR from cardiac procedures does not contribute the CHD-Cancer association. I would suggest that the authors refrain from including this sub-group analysis, which might question the internal validity, and to remove the unfounded statement on LDIR.

Reference

Lévesque, L. E., Hanley, J. A., Kezouh, A., & Suissa, S. (2010). Problem of immortal time bias in cohort studies: example using statins for preventing progression of diabetes. BMJ: British Medical Journal, 340(7752), 907-911. http://www.jstor.org/stable/40701710


**Response:** We thank their reviewer for raising this valid consideration. We agree that, while management guidelines regard 10–15 years as the generally agreed-upon induction period needed for solid tumors such as lymphomas to appear (3), there might be some uncertainty around this number. As such, we have now removed the sub-group analysis.

**Reviewer #3:**

The authors have addressed my concerns and I now recommend publication


**Response:** We wish to thank the reviewer once again for the valuable comments and points raised. We greatly appreciate the positive assessment of our revised manuscript.

**References:**

1.	Ahrensberg JM, Fenger-Gron M, Vedsted P. Use of primary care during the year before childhood cancer diagnosis: a nationwide population-based matched comparative study. PLoS One. 2013;8(3):e59098.

2.	Ahrensberg JM, Fenger-Gron M, Vedsted P. Primary Care Use before Cancer Diagnosis in Adolescents and Young Adults - A Nationwide Register Study. PLoS One. 2016;11(5):e0155933.

3.	Lui GK, Saidi A, Bhatt AB, Burchill LJ, Deen JF, Earing MG, et al. Diagnosis and Management of Noncardiac Complications in Adults With Congenital Heart Disease: A Scientific Statement From the American Heart Association. Circulation. 2017;136(20):e348-e92.
