# Supplementary material for: The relationship between congenital heart disease and cancer in Swedish children: A population-based cohort study
Source: PLoS Med. 2022 Feb 25;19(2):e1003903. doi: 10.1371/journal.pmed.1003903 (PMC8880823; doi:10.1371/journal.pmed.1003903)
Supplement: Attachment — Submitted filename: R3_CHDcancer_editorial comments with responses.docx [file pmed.1003903.s013.docx]

We thank the editorial team for their insightful comments on our manuscript throughout this review process. Below, we have responded to their requests in this revision.

After reading through the manuscript again, we have also corrected a typo in line 200 (used to read “have”, now reads “has”).

**REQUESTS FROM THE EDITORS:**

Please update the bullet point at line 69 to ‘These results suggest an association between CHD and childhood cancer and have the potential to facilitate early cancer detection by increasing awareness amongst physicians.’

**Response:** We have revised the bullet point as requested.

Please provide the unadjusted comparisons as well as the adjusted comparisons in Table 2.

**Response:** Table 2 now also includes the initial unadjusted model (which was previously omitted, due to the unadjusted estimates being fairly similar to those in Model 1).
