# Supplementary material for: The relationship between congenital heart disease and cancer in Swedish children: A population-based cohort study
Source: PLoS Med. 2022 Feb 25;19(2):e1003903. doi: 10.1371/journal.pmed.1003903 (PMC8880823; doi:10.1371/journal.pmed.1003903)
Supplement: Attachment — Submitted filename: CHDcancer_editorial&reviewer comments with responses.docx [file pmed.1003903.s011.docx]

We are very grateful for the valuable comments and suggestions for revision we received from the reviewers and the editorial board on the manuscript entitled “The relationship between congenital heart disease and cancer in Swedish children: a population-based cohort study” (PMEDICINE-D-21-02870R1) for consideration at PLOS Medicine. We believe that this thorough feedback helped us to substantially improve our paper and hope that you will find the revised manuscript suitable for publication. Our responses regarding how the comments from the Editors and Reviewers have been addressed are in blue font below.

**REQUESTS FROM THE EDITORS:**

🡪Please update your title to ‘The relationship between congenital heart disease and cancer in Swedish children: a population-based cohort study’.

**Response:** We have revised the title as requested.

🡪Data Availability Statement (DAS) requires revision:

•	Data availability statement: Please replace the word ‘secrecy’ with ‘privacy’.

•	A study author cannot be the contact person for the data.

**Response:** We have revised the Data Availability Statement, which now reads:

*“Anonymized personal data were obtained from national Swedish Registry holders after ethical approval and privacy assessment. According to Swedish laws and regulations, personal sensitive data can only be made available for researchers who fulfill legal requirements for access to personal sensitive data. Eligible individuals can apply for the data from the National Board of Health and Welfare in Sweden (*[*https://www.socialstyrelsen.se/statistik-och-data/bestalla-data-och-statistik/*](https://www.socialstyrelsen.se/statistik-och-data/bestalla-data-och-statistik/)*) and from Statistics Sweden (*[*https://www.scb.se/vara-tjanster/bestall-data-och-statistik/*](https://www.scb.se/vara-tjanster/bestall-data-och-statistik/)*).*

🡪Please structure your abstract using the PLOS Medicine headings (Background, Methods and Findings, Conclusions).

•	Please combine the Methods and Findings sections into one section, “Methods and findings”.

•	In the last sentence of the Abstract Methods and Findings section, please describe the main limitation(s) of the study's methodology.

**Response:** We have revised the structure of the abstract according to the PLOS Medicine headings, and added the following sentence at the end of the Abstract “Methods and Findings” section:

*“Limitations of the study include the National Patient Register not being nation-wide until 1987, in addition to the rareness of the conditions under study providing limited power for analyses on the rarer cancer subtypes.”*

🡪Please remove the ‘Financial disclosures’ from the title page. This information is captured as metadata based on your responses to the submission form.

**Response:** The title page has been updated as suggested.

🡪At this stage, we ask that you include a short, non-technical Author Summary of your research to make findings accessible to a wide audience that includes both scientists and non-scientists. The Author Summary should immediately follow the Abstract in your revised manuscript. This text is subject to editorial change and should be distinct from the scientific abstract. Please see our author guidelines for more information.

**Response:** We have now included the following Author Summary immediately following the abstract:

***Why was this study done?***

- *Birth defects have consistently been associated with an increased childhood cancer risk, although for congenital heart disease (CHD) the associations have been conflicting.*
- *Increasing the understanding of which birth defects predispose towards specific cancer types is key to carrying out targeted interventions for earlier cancer diagnosis.*
- *Children with CHD are living longer than ever before, therefore insights into the relationship with cancer are particularly compelling.*

***What did the researchers do and find?***

- *By combining high-quality Swedish health and population registers, 4,178,722 children born between 1973 and 2014 were included in this study— including 68,943 children with CHD.*
- *The findings showed that children born with CHD were at increased risk of lymphoma and hepatoblastoma, whereas an initially observed increased risk of leukemia was explained by Down syndrome.*
- *The risk of lymphoma was particularly elevated in children with complex CHD.*

***What do these findings mean?***

- *The results may contribute to an increased awareness of the predisposition to cancer in children with CHD, facilitating early cancer detection which may lead to better cancer outcomes.*
- *Further research should focus on the potential causal mechanisms underlying these findings, including genetics and radiation from cardiac procedures.*

🡪Please update your citations throughout: these should be in square brackets.

**Response:** The citations have been updated as suggested.

🡪Lines 159 and 161: PLOS does not permit "data not shown.” Please remove this claim, or if you are the owner of the data relevant to this claim, please provide the data in accordance with the PLOS data policy, and update your Data Availability Statement as needed.

**Response:** We appreciate the need to support these claims with the relevant data. As such, three new supplementary tables have been added: S3 Table includes the adjusted HRs of cancer in children with CHD in the subset of the population where we have data for maternal smoking (from 1982 onwards), S4 Table includes the adjusted HRs of cancer in children with CHD born during the period when the patient register achieved nation-wide coverage (from 1987 onwards), and S5 Table includes the stratified analysis by age at cancer diagnosis.

🡪Please refer to your study as a population-based cohort study in the Methods and Abstract.

**Response:** The text has been changed as suggested.

🡪Line 249: Please update to ‘To our knowledge, this is the largest study to date on cancer risk among children with CHD in a European population’.

**Response:** The text has been updated as suggested.

🡪Please discuss the risk of selection bias that could have been introduced by the exclusion of subjects with missing data.

**Response:** We concur that this is an important point to discuss and wish to thank the editors for bringing it up. Taking also into consideration the advice from Reviewer 2 in their 5^th^ point, we have now included a supplementary Table (S2 Table) with the distribution of the exposure and outcomes under study based on status of information on the main covariates. The table is referenced in the Methods section (line 169), and a section on selection bias has been added in the Discussion (lines 314–318):
*“Further, the exclusion of individuals with missing information on the main covariates, albeit very small (2.6%), may potentially have introduced some selection bias. However, the distribution of both CHD and cancer was very similar in children with missing and complete information on covariates, rendering such an eventuality unlikely.”*

🡪Please ensure that the study is reported according to the STROBE guideline, and include the completed STROBE checklist as Supporting Information. Please add the following statement, or similar, to the Methods: "This study is reported as per the Strengthening the Reporting of Observational Studies in Epidemiology (STROBE) guideline (S1 Checklist)."

When completing the checklist, please use section and paragraph numbers, rather than page numbers.

**Response:** The STROBE checklist has now been included as Supporting Information (S1 STROBE Checklist). The suggested statement has been added to the Methods section, lines 102–103.

🡪Did your study have a prospective protocol or analysis plan? Please state this (either way) early in the Methods section.

**a)** If a prospective analysis plan (from your funding proposal, IRB or other ethics committee submission, study protocol, or other planning document written before analyzing the data) was used in designing the study, please include the relevant prospectively written document with your revised manuscript as a Supporting Information file to be published alongside your study, and cite it in the Methods section. A legend for this file should be included at the end of your manuscript.

**b)** If no such document exists, please make sure that the Methods section transparently describes when analyses were planned, and when/why any data-driven changes to analyses took place.

**c)** In either case, changes in the analysis-- including those made in response to peer review comments-- should be identified as such in the Methods section of the paper, with rationale.

**Response:** We appreciate the importance of clarifying that analyses were defined a priori. We conducted our analyses according to a prospectively-written study protocol, that has now been included as a Supporting Information (S1 Analysis Plan) and is referenced in the start of the Methods section (lines 104–106). In addition to the analyses included in that, we evaluated the relationship between CHD complexity to further challenge our findings. We also included analyses of two additional cancer subtypes: hepatoblastoma and neuroblastoma, because a recent study [1] found particularly strong associations for these cancer types.

**COMMENTS FROM THE REVIEWERS:**

**Reviewer #1:**

The authors exanimated relationship between congenital heart disease (CHD) and childhood cancer risk. They aimed to determine the relationship between CHD and cancer in Swedish children. All individuals registered in the Swedish Medical Birth Register between 1973 and 2014 were included in the study. Individuals with CHD were identified from the MBR and National Patient Register, whereas cancer diagnoses were retrieved from the Swedish Cancer Register. The authors observed increased risks of childhood cancer overall, leukemia, lymphoma, and hepatoblastoma in children with CHD, but after adjustment for Down syndrome, only the increased lymphoma and hepatoblastoma risk remained. When restricting to CHD diagnoses from the MBR only, i.e., those diagnosed around birth, the risk for childhood cancer overall) and leukemia was more pronounced, even after controlling for Down syndrome. Moreover, they observed substantially elevated lymphoma risk in children with

complex CHD. In conclusions: they found associations between CHD and childhood lymphomas and hepatoblastomas, not explained by a diagnosis of Down syndrome. They observed stronger associations in complex CHD.

The study was perfectly designed and conducted.

**Response:** We thank the reviewer for this positive and highly encouraging assessment of our manuscript— we were very pleased to read that the reviewer was overall satisfied with the manuscript.

**Reviewer #2:**

**Summary:** This is a retrospective population-based cohort study conducted in order to determine the association between CHD and childhood cancer incidence among Swedish children. Using Medical Birth Registry and National Patient Register, the study identified over 68,000 CHD patients among nearly 4,171,000 newborn children between 1973 to 2014. Childhood cancers were retrieved by linking Swedish Cancer Registries. The associations were assessed using Cox’s proportional hazards model adjusting for birth decade, birth weight for gestational age, maternal/paternal age and education, region of residence at birth, neurocutaneous syndromes and/or Down syndrome.

**Overall comments:** Several other studies reported an elevated risk of cancer among both children and adults with CHD. However, the underlying mechanism is poorly understood. This study also provided evidence in favour of increased childhood cancer risk among children with CHD but as such, did not provide significant new information.

The strength of the study is the nationally representative large database spanning over 40 years of observation period. The objective of the study is clearly mentioned. However, with multiple sub-analyses, sensitivity analyses and outcomes, the communication and interpretability of results necessarily becomes challenging for the readers to follow.

Mentioning general and specific objectives with support of rationale could have been helpful. The study design and statistical methods are appropriate. However, there are some major concerns that need to be addressed:

**Response:** We thank the reviewer for bringing up the important discussion about the novelty of the study. While previous research has also focused on the relationship between CHD and childhood cancer, there is a distinct lack of large, population-based material with a long follow-up focusing on these rare defects. As findings from previous studies have been conflicting and often pertain to a wide age of ranges without considering childhood cancer separately, we believe that this large, population-based cohort with over 40 years of follow-up is an important addition to the knowledge base.

We also appreciate that the plethora of sub-analyses and sensitivity analyses might be challenging for the readers to follow; to hopefully aid with the understanding of the rationale behind these analyses, we have now added some explanatory sentences in the methods section, lines 154–164 (newly added parts in bold):

*“Subsequently, we also investigated CHD complexity, based on the categorization denoted above,* ***to investigate if associations were particularly pronounced among children with more severe CHD.****”*

*“Finally, separate sensitivity analyses were performed: first including maternal smoking in the models* ***to investigate whether it might drive some of the associations*** *(data only available in the MBR from 1982 onwards) …”*

*“Third, we stratified by age at cancer diagnosis (<10 years or ≥10 years)* ***to gauge if cancer risk might differ considerably between childhood and adolescence,*** *and finally we restricted to the years when the Patient Register had achieved nation-wide coverage of in-patient care (1987–2015)* ***to allow for better detection of CHD not diagnosed at birth.****”*

We were pleased to read that the reviewer found the overall study design and statistical methods appropriate for the research question. We also wish to thank the reviewer for the well-thought out and thorough points raised below; our point-by-point responses follow:

1. **Potential immortal time bias?** The individuals not diagnosed with CHD at birth were identified as CHD (exposed) using National Patient Register (NPR) on a later date after birth. Since the authors followed the final study population from birth, the time between the cohort entry and CHD diagnosis became ‘immortal’ for the exposed children; they cannot have cancer by design to have being classified as exposed. Did the author consider time-dependent exposure definition in this regard?

**Response:** We thank the reviewer for this important comment. We acknowledge that not being able to capture CHD at birth for all children can be a challenge in need of thorough consideration. We agree with the reviewer that using time-dependent exposure definition could have advantages; we believe that the advantages are similar to our current approach. When considering a time-dependent definition of the exposure, if the cancer occurred before the CHD diagnosis, follow-up would stop at the time of the cancer diagnosis and the child would be classified as an unexposed cancer case (since the CHD diagnosis had not yet occurred). In our approach, we also classified all children that had a cancer diagnosis before their CHD diagnosis as unexposed children. We believe that the two approaches both handle the most important risk of bias: detection/surveillance bias. Detection bias would occur if CHD was defined using all CHD diagnoses at any point in time, which would likely cause an overestimation of the association, as mild CHD might be detected among cancer cases precisely because of the cancer diagnosis, but not among the cancer-free population.

2. **2-year lag time:** The authors used 2 years of lag time for the subjects who were not diagnosed as a CHD patient at birth in order to account for surveillance bias. How about for the patients who were diagnosed at birth? Did they also apply lag time for them? If not, what were the assumptions and how are they not subject to such bias?

**Response:** We wish to thank the reviewer for this thoughtful comment. We would like to clarify that the lag period of 2 years was introduced with regards to the exposure (congenital heart disease diagnosis), rather than the outcome. As childhood cancer is generally well-diagnosed in Sweden [2], we believe that it would eventually be diagnosed regardless of CHD status, even if there was some delay. On the other hand, we were concerned that some of the milder CHD not diagnosed at birth might have remained undiagnosed, if not for the increased monitoring in relation to the cancer diagnosis (see response to comment 1). This is discussed in the Discussion section, lines 308–314.

3. **Adjusting for competing risk of death:** The analyses did not account for competing risk of death. Even though the mortality rate might be lower in this pediatric population, adjusting for death might account for overestimation of the association reported.

**Response:** We thank the reviewer for this remark. We wish to point out that indeed, there might be some cases where children diagnosed with CHD may die before a cancer can be diagnosed— we expect, however, that this would lead to an underestimation of the association of CHD with childhood cancer, rather than an overestimation. It should be noted that when competing events are infrequent, as is the case here, competing risk analyses are expected to yield similar results.

4. **Adjusting for CHD severity:** The authors presented CHD severity-stratified analyses but did not account for it in other models. More specifically, the individuals diagnosed as CHD patients at births are those with more severe lesions. Not controlling for them in the combined cohort might represent a spurious association.

**Response:** We thank the reviewer for bringing up CHD severity. We would like to clarify that the aim of the initial analysis (presented in Table 2) was to examine childhood cancer risk in any CHD, regardless of the severity of the underlying condition. In addition, we wanted to stratify for CHD severity, which is done in Table 3 (where children with mild-moderate CHD and severe CHD are compared to children without CHD). In order to present the latter analysis in a clearer way, we have now added that children without CHD are the reference group in Table 3. We have also added a clarification with regards to the rationale behind this analysis in the Methods section, lines 155–156:

*“…to investigate if associations were particularly pronounced for children with more severe CHD.”*

5. **Dealing with Missing data:** The authors excluded individuals with missing covariates (2.6%). However, they did not give any detail on the characteristics of these missing subjects: Are they more severe than those included in the final cohort or otherwise? What is the distribution of the primary endpoints among them? Are they risking any selection bias?

**Response:** We agree with the concerns raised by the reviewer and wish to thank them for pointing this out. Even though the register-based design allowed us to keep the proportion of individuals with missing data on covariates very low, describing their characteristics provides important information for the readers. We have now included a table in the supplement, S2 Table, including the distribution of characteristics/events of interest among these individuals and the study population. A reference to this table is in the Methods section (line 174), and the possibility of selection bias is discussed in the Discussion (lines 314–318):
*“**Further, the exclusion of individuals with missing information on the main covariates, albeit very small (2.6%), may potentially have introduced some selection bias. However, the distribution of both CHD and cancer was very similar in children with missing and complete information on covariates, rendering such an eventuality unlikely.”*

6. **Medical Comorbidities:** The authors did not report or adjusted for any medical comorbidities. More severe CHD patients might have more medical comorbidities and would lead to increased surveillance. Do the authors consider adjusting for them?

Response: We thank the reviewer for this thoughtful remark and agree that it might be likely that patients with more severe CHD also have other comorbidities and therefore could be closely monitored with regard to childhood cancer. However, as CHD is present from birth, we would consider comorbidities to be mediators of the association, rather than confounders— and they were therefore not included in the regression models. To address this concern, we have now added the following sentence in the limitations section of the discussion (lines 321–323):

*“Similarly, comorbidities that might occur to a larger extent in severe CHD and lead to increased cancer surveillance would also be mediators of the association.”*

7. **Sex as EMM or Confounder:** The authors adjusted for Sex in their models and also reported the sex-stratified hazard ratios. This reviewer does not find it informative in a situation when the manuscript did not discuss on the association by sex.

**Response:** We thank the reviewer for this remark and agree that there should be some discussion around the sex-stratified analyses. To address this, we have now added the following sentences:

*“Sex-stratified analyses showed that the increased risk for lymphoma was primarily observed among boys (HR: 1.83; 95% CI, 1.14–2.92), whereas the increased hepatoblastoma risk was seen in both boys (HR: 3.77; 95% CI, 1.37–10.37) and girls (HR: 3.93; 95% CI, 1.22–12.68).”* In the results section, lines 192–195.

*“The increased risk of lymphomas seemed to predominantly stem from non-Hodgkin’s lymphoma (HR 1.98; 95% CI, 0.98–4.01), which, as in overall lymphoma, was more pronounced in boys (HR 2.21; 95% CI, 0.98–4.99).”* In the results section, lines 212–215.

8. **Birth weight, Age categorized:** The authors represented and adjusted for birth weight for gestational age and maternal/paternal age as categorical variable. How did they choose the non-linear representation of the confounders over linear one? Did they perform any model comparison (eg. LRT)? They did not mention possibilities of residual confounding in this regard or any other sources (eg. omitting a very important confounder due to data limitation) in their discussion.

**Response:** We thank the reviewer for this consideration. As suggested in point 9c, we are no longer considering birth weight for gestational age as a covariate in our models. Maternal/paternal age were categorized according to common practices in cancer epidemiology. However, after reading this point along with the feedback from reviewer 3, we further considered parental age as a continuous variable. We also fitted models where we included the covariate as a quadratic polynomial term, to account for non-linear relations with the outcomes— finally, we considered fitting splines. We observed no difference after fitting the models thusly and have therefore retained our original categorization.

We would also like to clarify that the reason why residual confounding is not discussed is that very few risk factors are known for childhood cancer [3], and some of them are not related with CHD occurrence. To ensure that the manuscript better reflects this, the following sentence has been added to the Discussion Section, lines 323–325:
*“**Finally, the only other well-established risk factors for childhood cancer —prior chemotherapy and high-dose ionizing radiation— cannot predate the occurrence of CHD and therefore cannot be considered as potential confounders.”*

9. **Reporting:** This reviewer would like to mention some reporting issues that might be helpful to the authors:
   a. The authors might consider including a flowchart of derivation of the study population.

**Response:** We thank the reviewer for this suggestion and agree that such a flowchart can be helpful for the readers. As such, we have now included this flowchart as a supplementary figure (S1 Figure). A reference to that figure has been added in the Methods section, in the description of the study population (line 110).

b. Since this study relies on the time to event analyses, reporting follow-up times (median), total person-time etc. would be informative and helpful to understand the cohort.

**Response:** We thank the reviewer for this suggestion; we also believe that it can aid the readers in achieving a better understanding of the cohort. We have thus included the following sentence at the start of the Results section (lines 179–180):

*“This study included 4,178,722 children accumulating a total of 64,642,606 person-years of follow-up time (median follow-up: 15.1 years).”*

c. In Method section (Line 128), the author listed the potential confounders or *mediators*. From a theoretical and analytical point of view, we should not adjust for mediators. The authors might consider making correction for that.

**Response:** We thank the reviewer for this helpful suggestion and concur that not adjusting for mediators is indeed more appropriate. As such, birth weight for gestational age has been removed from all analyses.

d. For binary variable (eg. yes/no), reporting proportions for both categories might seem redundant to some of the readers. The authors might consider reporting only one (eg. yes)

**Response:** We agree that this might be a more appealing presentation of the baseline characteristics reported in Table 1 and are now reporting only the numbers and percentages for children with neurocutaneous syndromes and Down syndrome.

e. In the discussion section, the authors mentioned that their sub-analyses by age at cancer diagnosis did not support the hypothesis that exposure to LDIR from cardiac procedure explains the CHD-Cancer association. However, the data were not provided. This reviewer finds it necessary to provide the data in support to their claim.

**Response:** We thank the reviewer for this suggestion. We agree that it is better practice (and more helpful for the readers) to provide the data under question. As such, this data is now reported in the supplement, S5 Table.

**Reviewer #3:** I confine my remarks to statistical aspects of this paper. The general approach is fine, but I have some issues to resolve before I can recommend publication.

One big issue is that this is a population study -- that is, you have an entire population, not a sample. Many statisticians, including me, think that this makes inferential statistics odd. You don't have any inference to do, you have the whole population. Other statisticians say inference is still meaningful if you posit a sort of super-population (e,g, all Scandinavian children, all children anywhere ....) I'm not a big fan of this, but I wouldn't reject an article that did it. But it should be discussed. The authors (wisely, in my view) did not include p values but they did include confidence intervals. These have the same problem. I'd prefer leaving those out and putting in standard errors of the estimates.

**Response:** We thank the reviewer for bringing forth this perspective, as this is indeed a valuable discussion. Despite having information on what amounts to almost the entire Swedish population during the study period, we believe that this could still be regarded as a sample in space and time. Therefore, questions can still arise about the generalizability to other populations and time points. As such, we think that reporting confidence intervals is warranted in this case. To better reflect our aim of generalizing the findings to other populations, we have now added the following sentence in the Discussion section, lines 292–294:

*“As such, we believe the data in this study to be representative of the Swedish population— and the findings generalizable to similar populations.”*

On p. 6, lines 129-132, the authors categorize many continuous variables. This is nearly always a mistake. I wrote an article about this: but, briefly, leave the variables as is and use splines to investigate nonlinearity.

**Response:** We thank the reviewer for this consideration. The choice of categories was based on common practices in cancer epidemiology (and common practices in register-based epidemiology overall). We did, however, after reading this feedback further consider the variables as continuous, also fitting models where we included the continuous covariates as quadratic polynomial terms, to account for potential non-linear relations with childhood cancer— finally, we considered fitting splines. The findings remained virtually unchanged after fitting the models thusly: most point estimates and confidence intervals remained the same, with the largest difference observed in hepatoblastoma [fully adjusted HR with continuous variables fitted with a spline effect: 3.85(1.79–8.28); original fully adjusted HR: 3.83 (1.78–8.22)]. We have therefore retained our original categorization.

We also wish to mention that the variables in question were not associated with the outcomes (overall childhood cancer and cancer subtypes) in our data, so the different approaches of modelling these covariates are unlikely to change the association between CHD and childhood cancer. We have, however, retained them in our models because they theoretically could be confounders, and we had a priori defined them as such.

**References:**

1.	Lupo PJ, Schraw JM, Desrosiers TA, Nembhard WN, Langlois PH, Canfield MA, et al. Association Between Birth Defects and Cancer Risk Among Children and Adolescents in a Population-Based Assessment of 10 Million Live Births. JAMA Oncol. 2019. Epub 2019/06/21. doi: 10.1001/jamaoncol.2019.1215. PubMed PMID: 31219523; PubMed Central PMCID: PMCPMC6587148.

2.	Ward ZJ, Yeh JM, Bhakta N, Frazier AL, Atun R. Estimating the total incidence of global childhood cancer: a simulation-based analysis. Lancet Oncol. 2019;20(4):483-93. Epub 2019/03/03. doi: 10.1016/S1470-2045(18)30909-4. PubMed PMID: 30824204.

3.	Spector LG, Pankratz N, Marcotte EL. Genetic and nongenetic risk factors for childhood cancer. Pediatr Clin North Am. 2015;62(1):11-25. Epub 2014/12/02. doi: 10.1016/j.pcl.2014.09.013. PubMed PMID: 25435109; PubMed Central PMCID: PMCPMC4384439.
